# Extracellular CIRP Induces an Inflammatory Phenotype in Pulmonary Fibroblasts *via* TLR4

**DOI:** 10.3389/fimmu.2021.721970

**Published:** 2021-07-23

**Authors:** Siavash Bolourani, Ezgi Sari, Max Brenner, Ping Wang

**Affiliations:** ^1^ Center for Immunology and Inflammation, Feinstein Institutes for Medical Research, Manhasset, NY, United States; ^2^ Elmezzi Graduate School of Molecular Medicine, Manhasset, NY, United States; ^3^ Department of Surgery, Donald and Barbara Zucker School of Medicine at Hofstra/Northwell, Manhasset, NY, United States; ^4^ Department of Molecular Medicine, Donald and Barbara Zucker School of Medicine at Hofstra/Northwell, Manhasset, NY, United States

**Keywords:** fibroblast, eCIRP, inflammation, fibrosis, bleomicyn

## Abstract

Extracellular cold-inducible RNA-binding protein (eCIRP), a new damage-associated molecular pattern (DAMP), has been recently shown to play a critical role in promoting the development of bleomycin-induced pulmonary fibrosis. Although fibroblast activation is a critical component of the fibrotic process, the direct effects of eCIRP on fibroblasts have never been examined. We studied eCIRP’s role in the induction of inflammatory phenotype in pulmonary fibroblasts and its connection to bleomycin-induced pulmonary fibrosis in mice. We found that eCIRP causes the induction of proinflammatory cytokines and differentially expression-related pathways in a TLR4-dependent manner in pulmonary fibroblasts. Our analysis further showed that the accessory pathways MD2 and Myd88 are involved in the induction of inflammatory phenotype. In order to study the connection of the enrichment of these pathways in priming the microenvironment for pulmonary fibrosis, we investigated the gene expression profile of lung tissues from mice subjected to bleomycin-induced pulmonary fibrosis collected at various time points. We found that at day 14, which corresponds to the inflammatory-to-fibrotic transition phase after bleomycin injection, TLR4, MD2, and Myd88 were induced, and the transcriptome was differentially enriched for genes in those pathways. Furthermore, we also found that inflammatory cytokines gene expressions were induced, and the cellular responses to these inflammatory cytokines were differentially enriched on day 14. Overall, our results show that eCIRP induces inflammatory phenotype in pulmonary fibroblasts in a TLR4 dependent manner. This study sheds light on the mechanism by which eCIRP induced inflammatory fibroblasts, contributing to pulmonary fibrosis.

## Introduction

Extracellular cold-inducible RNA-binding protein (eCIRP), a novel damage-associated molecular pattern (DAMP) discovered by our lab, plays a key role in sepsis, hemorrhagic shock, ischemia-reperfusion injuries (1–5). eCIRP induces these effects *via* its binding to and activation of the toll-like receptor 4 (TLR4) and myeloid differentiation factor 2 (MD-2) receptor complex (6). The role of eCIRP in the activation of pulmonary cell populations such as macrophages (1), alveolar epithelial cells (7, 8), and endothelial cells (9) have been studied. However, the effect of eCIRP on pulmonary fibroblasts has yet to be studied.

Recently, we have discovered that eCIRP plays a role in the development of pulmonary fibrosis (publication forthcoming). Furthermore, a growing body of evidence suggests that blocking other DAMPs that also stimulate TLR4 can ameliorate pulmonary fibrosis (10–12). These factors have been shown to induce an inflammatory phenotype in fibroblasts through a TLR4 dependent process (13, 14) and that halting TLR4 dependent pathway alleviates the process of inflammatory fibroblast transformation (15–17). Fibroblasts are the key cells in the development of fibrosis in the lung. These mesenchymal cells are not terminally differentiated, and the principal regulator and potent inducer of fibroblast-to myofibroblast differentiation are transforming growth factor (TGF)-β (18–20). However, for persistent activation of fibroblasts to occur, TGF-β signaling needs to be enhanced by induction of a primed cellular microenvironment (21–24). A variety of signals and receptors have been shown to aid fibroblasts proliferation and cellular differentiation (25–29). However, the role of eCIRP on pulmonary fibroblasts has yet to be elucidated.

Inflammatory fibroblasts have been studied in various pathological processes such as neoplastic differentiation, autoimmune diseases, and fibrosis (30–32). Multiple factors have been implicated in the induction of inflammatory fibroblasts, such as mechanical stress, immunoglobulins, cytokines, and DAMPs (33–40). A growing body of evidence suggests that many factors that induce inflammatory fibroblast transformation operate in a TLR4 dependent manner (13–17). In this work, we examined the transcriptional response of pulmonary fibroblasts stimulated with eCIRP *in vitro*, focusing on proinflammatory, TLR4-induced pathways. We then evaluated how these pathways change over the course of bleomycin-induced pulmonary fibrosis.

## Materials and Methods

### Pulmonary Fibroblast Isolation and Culture

Wild type (WT) C57BL/6 mice and TLR4^-/-^ mice (Jackson Labs, on a C57BL/6 genetic background) were used in this study. All experiments involving live animals were carried out in accordance with the National Institutes of Health guidelines for the use of experimental animals (41) and were reviewed and approved by the Institutional Animal Care and Use Committee (IACUC) at the Feinstein Institutes for Medical Research. The lungs of 4-8-week-old WT and TLR4^-/-^ mice were explanted, and each group of pulmonary fibroblasts was isolated from a pool of 3-4 lungs, as previously described (42). The isolated fibroblasts were then cultured in DMEM supplemented with 10% heat-inactivated fetal bovine serum (FBS, MP Biomedicals), 2% penicillin-streptomycin, and L-glutamine (Gibco, ThermoFisher Scientific).

### *In Vitro* Stimulation With eCIRP and TGF-β1

At passages 3-6, the cultured medium was replaced with reduced serum media Opti-MEM. After incubation overnight in Opti-MEM, the pulmonary fibroblasts were treated with phosphate-buffered saline (PBS), 1 μg/mL recombinant mouse (rm) CIRP, 2 ng/mL rmTGF-β1 (R&D Systems), or rmCIRP plus rmTGF-β1. The rmCIRP was produced in our lab, as previously described (1). The cell lysates collected at 24 hours were used for high throughput mRNA sequencing and real-time reverse transcription-polymerase chain reaction (RT-PCR), the cell lysates collected at 48 hours were used for Western blotting (WB). Supernatants were collected at 48 hours and used for enzyme-linked immunosorbent assay (ELISA) quantification.

### Isolation of the mRNA, RNA-Seq, and Analysis

RNA was isolated using Ambion’s mirVana kit (catalog #AM1561, Thermo Fisher) following the manufacturer’s recommendations and quantified on a Nanodrop 2000 (catalog # ND-2000, Thermo Fisher). All samples had 260/280 and 260/230 ratios between 1.8 and 2.1. RNA quality was further assessed with an RNA Nano Chip using a Bioanalyzer 2100 (Agilent). All samples had a RIN number of 8 or higher. RNA was stored at -80 until Library preparation. Libraries were prepared using Illumina TruSeq Stranded Total RNA Library Prep Kit (catalog # RS-122-2203, Illumina Inc). The mRNA expression was obtained by RNA-Seq libraries run on NextSeq 550 following the manufacturer’s instructions. FastQC was used to ensure there was no adapter contamination and reads had acceptable quality scores (43). Sequenced segment alignments were performed using STAR2 (44) to the mouse GENCODE reference (45). The mRNA sequencing raw counts were obtained using ht-seq counts (46).

### MA Plots, Principal Component, Pathways, and Time Series Analysis

After high-throughput mRNA sequencing raw counts were obtained, the data were analyzed and normalized using the DESeq2 standard workflow (47). M (log ratio) and A (mean average) (MA) plots were obtained using the function *plotMA*, to visually represent the differences between PBS and rmCIRP-treated cells) by transforming the data onto logarithmic of base 2 (log_2_) fold changes *vs.* mean normalized count. The mRNA normalized counts were obtained using *plotCounts* function, which normalizes counts by sequencing depth. Principal component analysis was performed after variance stabilizing transformation (using *vst* function) to deal with the sampling variability of low counts and included correction for size factor (48). The pathway and time series analysis was done using the *Goexpress* workflow (49). The gene ontologies (GO) were first scored and ranked using the *GO_analyse* function, which consists of a random forest analysis to evaluate the ability of each gene to cluster samples according to the treatment groups in each analysis: pulmonary fibroblast mRNA sequencing data, PBS, eCIRP, TGF-β1, or eCIRP+TGF-β1; GSE132869 mRNA sequencing data, PBS, or bleomycin. The heatmap for each GO was created using the *heatmap_GO* function and Z-scores were scaled on each gene included in GO. Time-series data were analyzed using the *expression_plot* function, which plots the expression profile of a specific gene smoothed across time points (days 7, 14, 21, 28, and 42) while representing the mean and confidence interval of groups of lung tissue samples (bleomycin and PBS injected groups).

### Protein Detection and Quantification

For Western blotting, the cultured WT and TLR4^-/-^ primary pulmonary fibroblasts cells were scraped from 6 well culture plates and homogenized in a radioimmunoprecipitation assay buffer (RIPA) buffer containing phenylmethylsulfonyl fluoride (PMSF), Na-orthovanadate, and protease inhibitors. After centrifugation of cell lysates at 4°C and 12,000 rpm for 15 min, the total protein concentration was measured. The 10 μg protein of each sample was run in NuPAGE™ 4–12% Bis-Tris Gel (Invitrogen, Thermo Fisher Scientific), and gels were transferred to a nitrocellulose membrane. Membranes were blocked with 0.1% casein 1h at the room temperature, after that, they were incubated with primary antibodies such as CIRP (catalog # 10209-2-AP, Protein Tech), β-actin (clone AC-15, catalog # A5441, MilliporeSigma), tumor necrosis factor (TNF-α, catalog # 3707S, Cell Signalling Technology) and interleukin 1β (IL-1β, catalog #16806-1-AP, Protein Tech), MD2 (catalog # B100-56655, Novus Biologicals) and glyceraldehyde 3-phosphate dehydrogenase (GAPDH, catalog # 60004-1-lg, Proteintech) at 4°C overnight. After washing the membranes with Tris-Buffered Saline (TBS) containing 0.1% Tween-20 (TBST), and TBS; they were incubated with secondary antibodies for 1 h at room temperature. The signal of protein bands was detected and measured by using Odyssey CLx Imaging Machine and Image Studio Ver 5.2 Software. The bands were normalized with GAPDH. For ELISA, the levels of IL-6 of cell culture supernatant were measured using the mouse IL-16 DuoSet ELISA kit (DY206, R&D Systems), and it was performed according to the company’s protocol.

### Isolation and Analysis of RT-PCR

Total RNA was isolated using Trizol reagent (Invitrogen) and reverse transcribed to cDNA using M-MLV (Moloney murine leukemia virus) reverse transcriptase (Applied Biosystems, Foster City, CA). Polymerase chain reactions (PCR) were carried out in a final volume of 10.5 μl, which included a 5 μl SYBR Green PCR master mix (Applied Biosystems) and 31 nM of both reverse and forward primers. GAPDH mRNA was used to normalize the amplification data, and fold changes were calculated in comparison with WT Control mice using the 2^-(ΔΔCt)^ method. The sequence of forward and reverse primers used were as follows:

GAPDH: 5’-CATCACTGCCACCCAGAAGACTG-3’ (forward) and 5’-ATGCCAGTGAGCTTCCCGTTCAG-3’(reverse);
TNF-α: 5’-AGACCCTCACACTCAGATCATCTTC-3’ (forward) and 5’-TTGCTACGACGTGGGCTACA-3’ (reverse);
IL-1β: 5’-CAGGATGAGGACATGAGCACC-3’ (forward) and 5’-CTCTGCAGACTCAAACTCCAC-3’(reverse);
IL-6: 5’-CCGGAGAGGAGACTTCACAG-3’ (forward) and 5’-GGAAATTGGGGTAGGAAGGA-3’(reverse);
MD2 (Ly96): 5’-CGCTGCTTTCTCCCATATTGA-3’ (forward) and 5’-CCTCAGTCTTATGCAGGGTTCA-3’(reverse).

### Analysis of Gene Expression Omnibus Dataset

The expression datasets were was obtained directly from the gene expression omnibus website (https://www.ncbi.nlm.nih.gov/geo) using the *getGEO* function of R (http://www.r-project.org/) 4.0.3 library called *GEOquery* (50). After obtaining the dataset for GSE132869, the columns from lung tissues were selected. This GEO contains RNA sequenced from lung tissues of mice injected with bleomycin (10 μg/g/day, 100 μl of 1 mg/ml solution per injection of a ~20 g mouse) or PBS five times per week for two weeks. The tissues were collected on 7, 14, 21, 28, and 42 days after the first injection (51). While the exact timing of peak inflammation and fibrosis in the lung, in this model, is not clear, based on previous studies, inflammation occurs between days 7 and 14, and peak fibrosis likely happens between days 21 and 28 (52–54). Day 14, which we call the inflammatory-to-fibrotic transition phase of exposure to bleomycin, is a time when both processes are active.

### Statistical Analysis

Data from mRNA sequencing normalized count were analyzed using the results function of DESeq2 workflow, which estimates the log_2_ fold change of maximum likelihood estimates (MLE) between treated and untreated samples per unit of change on the specific genes, and p-values provided are Benjamini-Hochberg (BH)-adjusted p-values with false discovery cut-off set to 0.1 (55). The p-values are set to 0 if they are too small to be represented (the smallest floating-point value to be represented in R is 2.225074e-308). Data for RT-PCR, WB, and ELISA are shown in the bar graphs as mean and standard deviation (SD), with individual values depicted by colored points. The comparisons were performed using two-way analysis of variance (ANOVA) followed by Tukey’s multiple comparison test with the factors of two-way ANOVA being TLR4 gene expression status (WT *vs.* TLR4^-/-^) and fibroblast treatment (PBS *vs.* CIRP). The groups were checked for Shapiro-Wilk test for normality prior to the application of two-way ANOVA.

## Results

### eCIRP Induces Proinflammatory Cytokines in Pulmonary Fibroblasts *via* TLR4

**Figure 1:**
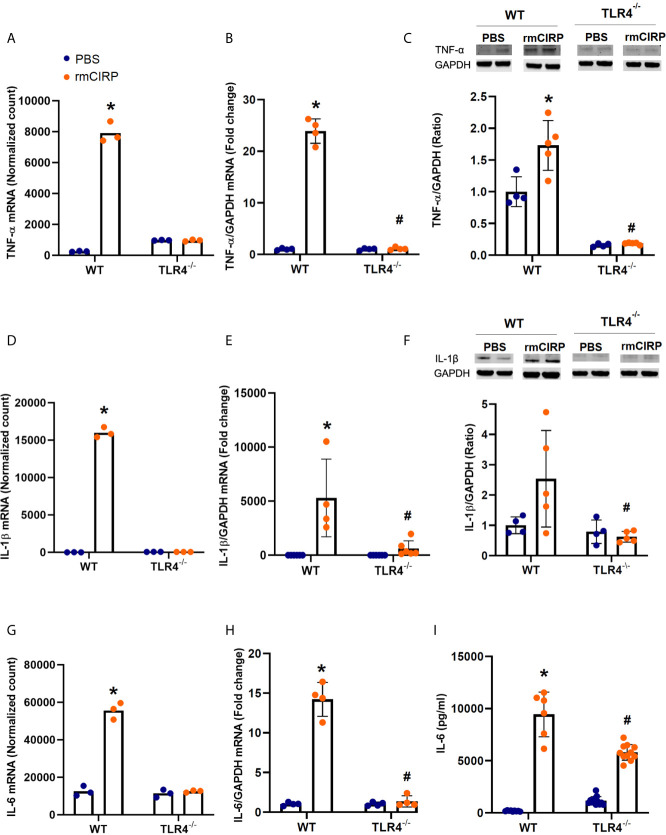
eCIRP induces proinflammatory cytokines in pulmonary fibroblasts in a TLR4-dependent manner. Differential mRNA expression normalized counts for TNF-α **(A)**, IL-1β **(D)**, and IL-6 **(G)** in pulmonary fibroblasts isolated from wild type (WT) and TLR4^-/-^ treated with PBS and 1 μg/ml CIRP. Real-time reverse transcription-polymerase chain reaction (RT-PCR) for TNF-α **(B)**, IL-6 **(E)**, and **(G)** normalized against Glyceraldehyde 3-phosphate dehydrogenase (GAPDH) mRNA. Western blot (WB) analyses of TNF-α **(C)** and IL-1β **(F)**, and enzyme-linked immunosorbent assay (ELISA) analysis of IL-6 **(I)** in WT and TLR4^-/-^ pulmonary fibroblasts treated with PBS and 1 μg/ml CIRP. Analyses for **(A, D, G)** are performed by log2 fold change of maximum likelihood estimates (MLE) using the DESeq2 workflow and the p-values were adjusted by Benjamini-Hochberg (BH) with a false discovery cut-off set to 0.1. Analyses for **(E, F, H, I)** were done using two-way ANOVA with one factor being genetics (WT *vs* TLR4^-/-^) and one factor being treatment (PBS *vs.* CIRP). *p<0.05 *vs.* WT PBS. ^#^p<0.05 *vs.* WT CIRP. The groups were checked for Shapiro-Wilk test for normality prior to the application of two-way ANOVA. The mRNA sequencing (3 samples per group, 3 repeats per sample) and RT-PCR (4 samples per group, 2 repeats per sample) were done after the cells were incubated for 24 hours. The WBs (4-5 samples per group) and ELISA (6-12 samples per group, triplicate) were performed when cells were incubated for 48 hours. Representative samples for each lineage (WT *vs.* TLR4^-/-^) are from a single blot shown for western blot analyses.

To examine the effects of eCIRP on the expression of proinflammatory cytokines, we examined the normalized mRNA count (RNA-Seq), RT-PCR expression, and supernatant protein levels of TNF-α, IL-1β, and IL-6 in pulmonary fibroblasts isolated from the lungs of WT and TLR4^-/-^ mice and cultured for 24h with rmCIRP or PBS. Compared with PBS, rmCIRP significantly increased the number of the mRNA normalized counts of TNF-α by 4.9 log_2_ fold (adjusted p-value**≈**0, **Figure 1A**), IL-1β by 9.9 log_2_ fold (adjusted p-value**≈**0, **Figure 1D**), and IL-6 by 2.1 log_2_ fold (adjusted p-value **=** 2.31e-33, **Figure 1G**). However, there were no significant increases in the mRNA normalized counts of TNF-α, IL-1β, and IL-6 in TLR4^-/-^ cells stimulated with rmCIRP as compared to PBS (**Figures 1A, D, G**; for more details, please refer to **Supplementary Table 1**). We confirmed the RNA-Seq results using RT-PCR amplification of mRNA obtained from a separate set of cells. Compared with PBS, rmCIRP significantly induced the WT fibroblast RT-PCR expression of TNF-α (23.6 fold, p<0.0001, **Figure 1B**), IL-1β (3368.7 fold, p= 0.0002, **Figure 1E**), and IL-6 (14.0 fold, p<0.0001, **Figure 1H**) expression by rmCIRP. The mRNA levels of TNF-α, IL-1β, and IL-6 measured by RT-PCR in TLR4^-/-^ pulmonary fibroblasts stimulated with rmCIRP, however, were significantly lower than those of WT stimulated with rmCIRP (**Figures 1B, E, H**). We also measured the effects of stimulation with rmCIRP on the pulmonary fibroblast protein expression of the above-mentioned cytokines (TNF-α and IL-1β by WB, IL-6 by ELISA). Compared with PBS, rmCIRP significantly induced the WT fibroblast protein expression of TNF-α (1.7 fold, p=0.0021, **Figure 1C**) and IL-6 (53.4 fold, p<0.0001, **Figure 1I**) expression by rmCIRP. The protein levels of TNF-α and IL-6 in TLR4^-/-^ pulmonary fibroblasts stimulated with rmCIRP, however, were significantly lower than those of WT stimulated with rmCIRP (**Figures 1C, I**). Stimulation with rmCIRP increased the protein expression of IL-1β in WT pulmonary fibroblasts by 2.5 fold, which showed a trend towards statistical significance (p=0.0884, **Figure 1F**). However, the protein expression of IL-1β in rmCIRP-stimulated TLR4^-/-^ pulmonary fibroblasts was 75% lower than that of rmCIRP-stimulated WT cells (p=0.0191, **Figure 1F**). Taken together, these results indicate that eCIRP induces proinflammatory cytokines in pulmonary fibroblasts in a TLR4 dependent manner.

### eCIRP Differentially Enriches TLR4 and its Dependent Pathways in Pulmonary Fibroblasts

**Figure 2:**
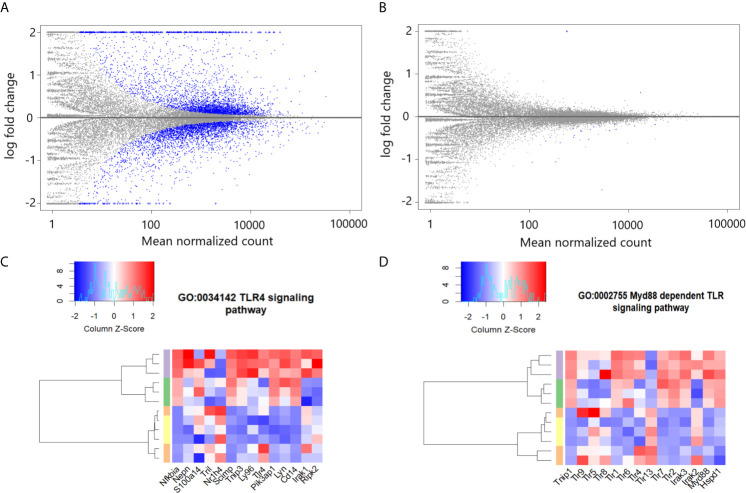
eCIRP differentially enriches TLR4 and dependent pathways in pulmonary fibroblasts irrespective of TGF-β1. MA plots for differential mRNA expression profile of 1 μg/ml CIRP treatment *vs.* PBS treatment of pooled pulmonary fibroblasts isolated from wild type (WT) **(A)** and TLR4^-/-^ mice **(B)**. Differential enrichment profile transcriptome of WT pulmonary fibroblasts isolated from mice and treated with PBS (orange), 2 ng/ml TGF-β1 (yellow), 1 μg/ml CIRP (purple), and the combination of the two (green) in gene ontologies 0034145 (toll-like receptor 4 signaling pathway) **(C)**, 0002755 (MyD88-dependent toll-like receptor signaling pathway) **(D)** shown in heatmaps. The dendrogram clustering was made based on the hierarchical clustering of samples across all genes for each ontology profile. The heatmap color spectra were normalized across each gene. Z-score color key and histogram of counts presented in the left upper corner. Pulmonary fibroblasts were collected 24 hours after treatments described and prepared for RNA sequencing.

eCIRP binds to and activates TLR4 (1). To determine the importance of TLR4 in eCIRP’s induction of proinflammatory pulmonary fibroblasts, we examined the MA plots of genomic data from high throughput sequencing of mRNA data in WT and TLR4^-/-^ pulmonary fibroblasts. Our analysis showed that the eCIRP-induced translational profile was restricted to WT pulmonary fibroblasts and did not occur in TLR4^-/-^ pulmonary fibroblasts (**Figures 2A, B**). In order to determine the effects of rmCIRP on the TLR4 and its related pathways, we analyzed the enrichment for transcripts associated with the TLR4 (GO:0034142) and Myd88 (GO:0002755) signaling pathways in WT pulmonary fibroblasts treated with PBS, rmCIRP, rmTGF-β1, or the combination of the rmCIRP and rmTGF-β1. Our analysis showed that stimulation with rmCIRP was associated with differential enrichment for both GO:0034142 and GO:0002755 (p-values; GO:0034142, <0.0001, **Figure 2C**; GO:0002755, 0.0007, **Figure 2D**), irrespective of the presence of TGF-β1. These results indicate that eCIRP changes the translational profile of pulmonary fibroblasts *via* TLR4 and induces TLR4-dependent pathways, irrespective of TGF-β1.

### eCIRP Induces MD2 Expression in Pulmonary Fibroblasts in a TLR4 Dependent Manner

**Figure 3:**
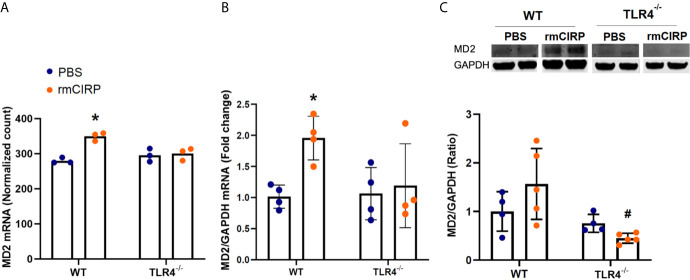
eCIRP induces MD2 expression in pulmonary fibroblasts in a TLR4 dependent manner. Differential mRNA expression profile **(A)**, real-time reverse transcription-polymerase chain reaction (RT-PCR) **(B)**, and western blot (WB) analyses for MD2 in WT and TLR4^-/-^ pulmonary fibroblasts treated with PBS and 1 μg/ml CIRP. Analysis for A was performed by log2 fold change of maximum likelihood estimates (MLE) using the DESeq2 workflow and the p-values were adjusted by Benjamini-Hochberg (BH) with a false discovery cut-off set to 0.1. Analyses for **(B, C)** were done using two-way ANOVA with one factor being genetics (WT *vs* TLR4^-/-^) and one factor being treatment (PBS *vs.* CIRP). *p<0.05 *vs.* WT PBS. ^#^p<0.05 *vs.* WT CIRP. The groups were checked for Shapiro-Wilk test for normality prior to the application of two-way ANOVA. The mRNA sequencing (3 samples per group, 3 repeats per instance) and RT-PCR (4 samples per group, 2 repeats per instance) were done after the cells were incubated for 24 hours. The WBs (4-5 samples per group) were performed when cells were incubated for 48 hours. Representative samples for each lineage (WT *vs.* TLR4^-/-^) are from a single blot shown for western blot analyses.

eCIRP also binds to MD2, which forms a complex with TLR4 (1). To examine the role of eCIRP in the induction of TLR4’s accessory pathway through the MD2 receptor, we examined the RNASeq normalized mRNA count, RT-PCR mRNA expression, and protein expression of MD2 in pulmonary fibroblasts isolated from the lungs of WT and TLR4^-/-^ mice. Stimulation with rmCIRP significantly increased the MD2 mRNA normalized count in WT cells by 0.3 log_2_ fold (adjusted p=0.004, **Figure 3A**). This induction, however, was not observed in the mRNA normalized count of TLR4^-/-^ cells (**Figure 3A**). Stimulation with rmCIRP also increased the mRNA expression of MD2 in WT pulmonary fibroblasts by 1.9 fold (p=0.047), but a similar increase was not seen in TLR4^-/-^ pulmonary fibroblasts (**Figure 3B**). Although there was an increase in protein expression of MD-2 in WT pulmonary fibroblasts treated with eCIRP, the increase was not statistically significant. Conversely, the MD2 protein expression of TLR4^-/-^ pulmonary fibroblasts stimulated with rmCIRP was significantly lower than that of WT pulmonary fibroblasts stimulated with rmCIRP (p=0.3741, **Figure 3C**). These data suggest that eCIRP induces MD2 expression in pulmonary fibroblasts in a TLR4 dependent manner.

### eCIRP Induction of Proinflammatory Pulmonary Fibroblast Phenotype Is Not Dependent on TGF-β1

**Figure 4:**
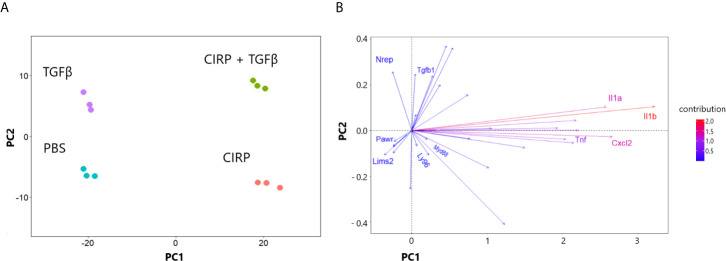
eCIRP induction of proinflammatory phenotype is not dependent on TGF-β1. Principal component analysis (PCA) of the mRNA expression profile of WT pulmonary fibroblasts treated with PBS, 1 μg/ml CIRP, 2 ng/ml TGF-β1, and combination of the two shown in clustering of samples **(A)** and direction of selected proinflammatory/profibrotic genes **(B)**. Pulmonary fibroblasts were collected 24 hours after treatments and prepared for RNA sequencing. PC1 and PC2 explained 84 and 11% of the variance, respectively. PC, Principal component.

In the previous section, we showed that TGF-β1 did not significantly affect the transcriptional profile of TLR4 and Myd88 signaling pathways in WT pulmonary fibroblasts (**Figures 2C, D**). To determine whether eCIRP requires TGF-β1 to induce the proinflammatory phenotype, we conducted a principal component (PC) analysis of mRNA profile sequenced from WT pulmonary fibroblasts treated with PBS, rmCIRP, rmTGF-β1, and the combination of the rmCIRP and rmTGF-β1. Our analysis showed that two PCs explain 95% of the variation in mRNA genomic data in WT pulmonary fibroblasts: PC1 explained 84% of the variance and PC2, 11%. Furthermore, the variance explained by PC1 aligned well with stimulation by rmCIRP, whereas PC2 aligned well with stimulation by TGF-β1 (**Figure 4A**). We then examined selected genes to see the direction of contribution and observed that TNF-α, IL-1α, IL-1β, IL-6, and CxCl2 contributed to the variance towards the eCIRP-aligned PC1. Furthermore, MD2 (Ly96) and Myd88 also contributed to the variance along PC1, albeit to a lesser degree (**Figure 4A**). To see the full list of genes examined, please see **Supplementary Figure 1**. Taken together, these results indicate that eCIRP induces a proinflammatory phenotype of pulmonary fibroblasts independently of TGF-β1.

### eCIRP Differentially Enriches Proinflammatory Transcriptional Profiles Irrespective of TGF-β1

**Figure 5:**
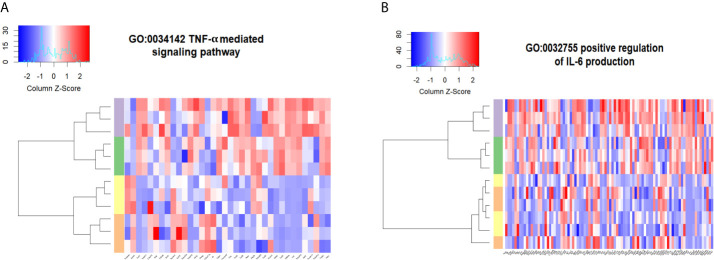
eCIRP differentially enriches proinflammatory transcriptional profiles irrespective of TGF-β1. Differential enrichment profile of WT pulmonary fibroblasts isolated from mice and treated with PBS (orange), 2 ng/ml TGF-β1 (yellow), 1 μg/ml CIRP (purple), and the combination of the two (green) in gene ontologies 0033209 (TNF-α signaling pathway) **(A)** and 0032755 (positive regulation of IL-6 production) **(B)** shown in heatmaps. The dendrogram clustering was made based on the hierarchical clustering of samples across all genes for each ontology profile. The heatmap of the color spectra was normalized across each gene. Z-score color key and histogram of counts presented in the left upper corner. Pulmonary fibroblasts were collected 24 hours after treatments described and prepared for RNA sequencing.

Stimulation with rmCIRP also caused a transcriptional reprogramming that was differentially enriched for genes associated with the TNF-α mediated signaling pathway (GO:0033209, p-value = 0.008, **Figure 5A**) and positive regulation of IL-6 production (GO:00032755, p-value <0.0001, **Figure 5B**) in WT pulmonary fibroblasts stimulated with rmCIRP or the combination of the rmCIRP and rmTGF-β1. However, differential enrichment for these two pathways was not present in the transcriptome of pulmonary fibroblasts cultured with rmTGF-β1. To see high-resolution figures to examine genes involved in GOs analyzed, please see **Supplementary Figures 2, 3**. As such, eCIRP differentially enriches the proinflammatory transcriptional profile in pulmonary fibroblasts that includes TNF-α signaling and IL-6 production.

### Proinflammatory Gene Profiles in Bleomycin Treated Lungs in the Inflammatory-To-Fibrotic Transition Phase Recapitulate Pulmonary Fibroblasts Stimulated With eCIRP

**Figure 6:**
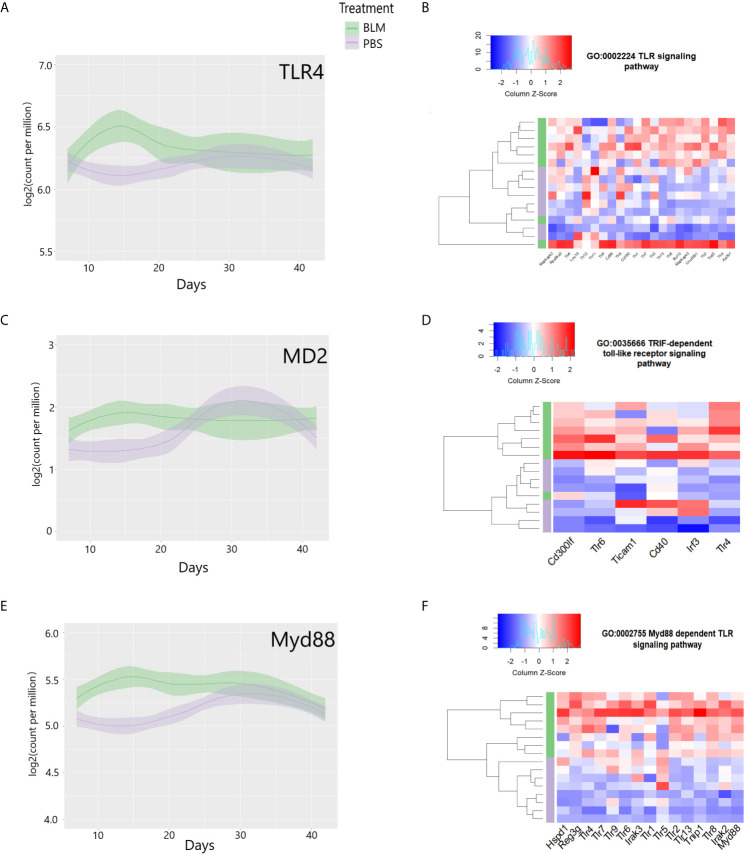
Toll-like receptor 4 (TLR4) and related genes are induced, and pathways are differentially enriched on day 14 of bleomycin injection in lung tissue. Examinations of a publicly available gene expression omnibus (GEO) profile: GSE132869. Expression profiles of TLR4 **(A)**, MD-2 **(C)**, and Myd88 **(E)** mRNA as base 2 logarithmic counts per million over time (days) in lung tissue of mice with the means and 0.95 confidence intervals. The samples are presented as bleomycin injected group (in green), and PBS injected mice (in purple). The plots are smoothed using a generalized linear model. Differential enrichment profile of lung tissues of PBS injected (purple) and bleomycin injected mice on day 14 from the start of injections in gene ontologies 0002224 (toll-like receptor signaling pathway) **(B)**, 0035666 (TRIF-dependent toll-like receptor signaling pathway) **(D)**, and 0002755 (MyD88-dependent toll-like receptor signaling pathway) **(F)** shown in the heatmap. The dendrogram clustering was made based on the hierarchical clustering of samples across all genes for each ontology profile. The heatmap of the color spectra was normalized across each gene. Z-score color key and histogram of counts presented in the left upper corner. Female mice (~12 weeks old) subcutaneously injected daily either with bleomycin (10 mg/kg/day) or PBS. Injections began on Day 0 and were done five times per week for two weeks. Mice were sacrificed on days 7, 14, 21, 28, and 42, and the lung tissues were collected.

To compare the transcriptomic changes after stimulation with eCIRP with those in the lungs of mice exposed to bleomycin, we examined a publicly available Gene Expression Omnibus (GEO) profile (GSE132869) containing high throughput RNA sequences from lung tissue of WT mice collected on days 7, 14, 21, 28, or 42 from the start of bleomycin injection. Similar to our observations in eCIRP-stimulated lung fibroblasts, the lung transcriptome on day 14 after bleomycin showed significant upregulation of TLR4, MD2, and Myd88 mRNA counts (**Figures 6A, C, E**). The lung transcriptome on day 14 was also differentially enriched for genes associated with the TLR signaling pathway (GO:0002224, p-value = 0.005, **Figure 6B**), TRIF dependent TLR signaling pathway (GO:0035666, p-value = 0.013, **Figure 6D**), and Myd88 dependent TLR signaling pathway (GO:0002755, p-value = 0.04, **Figure 6F**). To see a higher resolution of **Figure 6B**, please refer to **Supplementary Figure 4**. As a whole, the results indicate that the TLR4 and related genes and pathways activated by eCIRP in pulmonary fibroblasts are also transcriptionally active on day 14 after bleomycin injection.

**Figure 7:**
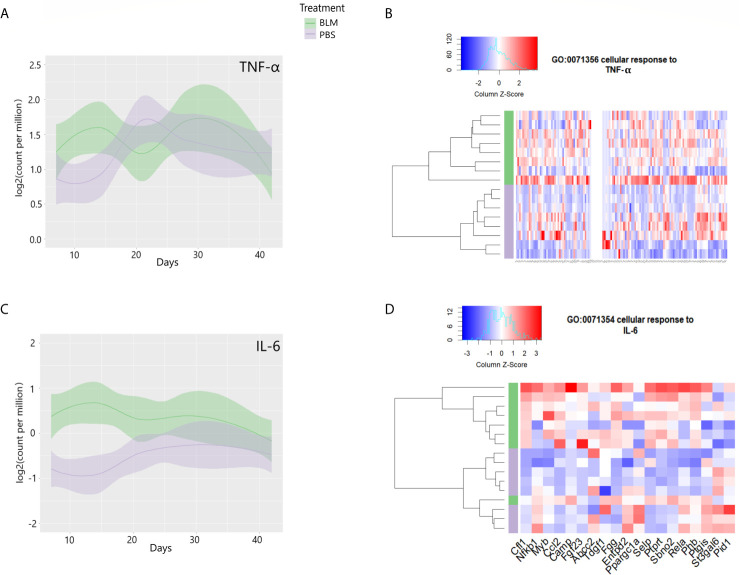
Proinflammatory cytokines are induced, and their cellular responses are enriched on day 14 of bleomycin injection in lung tissue. Examinations of a publicly available gene expression omnibus (GEO) profile: GSE132869. Expression profiles of TNF-α **(A)** and IL-6 **(C)** mRNA as base 2 logarithmic counts per million over time (days) in lung tissue of mice with the means and 0.95 confidence intervals. The samples are presented as bleomycin injected group (in green), and PBS injected mice (in purple). The plots are smoothed using a generalized linear model. Differential enrichment profile of lung tissues of PBS injected (purple), and bleomycin injected mice on day 14 from the start of injections in gene ontologies 0071356 (cellular response to TNF-α, **B**) and 0071354 (cellular response to IL-6, **D**) shown in the heatmap. The dendrogram clustering was made based on the hierarchical clustering of samples across all genes for each ontology profile. The heatmap of the color spectra was normalized across each gene. Z-score color key and histogram of counts presented in the left upper corner. Female mice (~12 weeks old) subcutaneously injected daily either with bleomycin (10 mg/kg/day) or PBS. Injections began on Day 0 and were done five times per week for two weeks. Mice were sacrificed on days 7, 14, 21, 28, and 42, and the lung tissues were collected.

To determine the timeline at which inflammatory cytokines and their cellular response pathways are induced, we examined the mRNA of cytokines TNF-α, IL-6, and the cellular response to these cytokines. Similar to our observations in eCIRP-stimulated lung fibroblasts, the lung transcriptome on day 14 after bleomycin showed significant upregulation of TNF-α and IL-6 mRNA counts (**Figures 7A, C**). The lung transcriptome on day 14 was also differentially enriched for genes associated with the cellular response to TNF-α (GO:0071356, p-value = 0.009, **Figure 7B**) and cellular response to IL-6 (GO:0071354, p-value= 0.021, **Figure 7D**). To see a higher resolution of **Figure 7B**, please refer to **Supplementary Figure 5**. Taken together, the results indicate that the TLR4 and related genes and pathways activated by eCIRP in pulmonary fibroblasts are also transcriptionally active on day 14 after bleomycin injection.

## Discussion

Fibroblasts are key cells involved in the fibrotic process (56). In order to illuminate the possible contribution of eCIRP to the fibrotic process, we investigated how eCIRP influences the transcriptional profile of the pulmonary fibroblasts. We evaluated the transcriptional response of lung fibroblasts stimulated with eCIRP with a particular focus on proinflammatory and TLR4-induced pathways. We also evaluated how these pathways change over the course of bleomycin-induced pulmonary fibrosis. What we observed was that eCIRP amplifies proinflammatory cytokines in a TLR4 dependent manner and differentially enriches the inflammatory pathways. Furthermore, we found that this activation pattern recapitulates that of the inflammatory-to-fibrotic transition phase in the bleomycin-induced pulmonary fibrosis model.

We have previously shown that CIRP binds with high affinity to the TLR4-MD2 complex, as well as to TLR4 and MD2 individually (1). Furthermore, TLR4 expression is known to be elevated in lung biopsies of patients with pulmonary fibrosis caused by chronic inflammatory diseases (21, 57, 58). While TGF-β1 is required for profibrotic activation of the fibroblasts, this activation and induction of organ fibrosis require a primed cellular microenvironment, which can be induced by TLR4 activation (22, 59, 60). The role of inflammatory fibroblasts in the pathogenesis of fibrosis has been studied (36, 61, 62). Inflammatory fibroblasts persistently activate the fibroblasts in an autocrine/paracrine manner in the lungs, resulting in pulmonary fibrosis (63–65). The proinflammatory role of TLR4-MD2 complex and Myd88 signaling pathway in the lungs has also been evaluated (66–69) and associated with the fibrotic process leading to pulmonary fibrosis (60, 70–74). In our study, we found that eCIRP induces proinflammatory cytokines and their pathways in pulmonary fibroblasts in a TLR4 dependent manner. Furthermore, we showed that the TLR co-receptor MD2 and the downstream Myd88 pathway are enriched by eCIRP in a TLR4 dependent manner. These results shed light on the mechanism by which eCIRP induces proinflammatory pulmonary fibroblasts during the inflammatory phase in the pathogenesis of bleomycin-induced pulmonary fibrosis.

It is well known that TGF-β1 is the master regulator, and its signaling is required for the promotion and maintenance of the fibrotic process in the lungs (75–77). The recent works by Bhattacharyya et al. showed that TLR4 signaling synergizes with TGF-β in the activation of fibroblasts in the setting of pulmonary fibrosis (21, 78, 79). After illustrating that eCIRP proinflammatory programming of fibroblasts is done in a TLR4 dependent, we set out to see the effect of TGF-β in this process. Our principal component analysis showed that the principal component that explains the variation in inflammatory cytokines and chemokines aligns with the treatment of eCIRP and not TGF-β1 (**Figure 4** and **Supplementary Figure 6**). This confirms that the proinflammatory programming of fibroblasts is dependent on eCIRP, not TGF-β1.

In order to establish how eCIRP’s induction of proinflammatory phenotype may contribute to the pathogenesis of bleomycin-induced pulmonary fibrosis, we examined the pathways induced by eCIRP in fibroblast in the lung tissues of mice. We showed that TLR4, MD2, Myd88, and related pathways are significantly induced in lungs collected on day 14 of bleomycin-induced pulmonary fibrosis. Proinflammatory cytokines and the related were also significantly overrepresented in day 14 of pulmonary fibrosis lung tissues, showing that the changes in the lung transcriptome during the inflammatory-to-fibrotic transition phase after bleomycin injection recapitulate those pulmonary fibroblasts stimulated with eCIRP. This is in line with previous studies showing an increase in the lung infiltration by total white blood cells, neutrophils, and macrophages peaks at day 14, the expressions of profibrotic markers peak at day 21-28 (53, 54, 80, 81). We have previously shown that eCIRP promotes acute lung injury *via* activation of macrophages, neutrophils, pneumocytes, and lung vascular endothelial cells in the contexts of sepsis, hemorrhagic shock, and intestinal ischemia/reperfusion injury (1, 5, 6, 82–84). However, little was known about eCIRP’s role in chronic inflammatory diseases of the lung. Our results show that eCIRP activates a proinflammatory phenotype in pulmonary fibroblasts, illuminating a potential mechanism by which eCIRP in the lung environment in the setting of chronic inflammation plays a critical priming role leading to lung fibrosis.

Our results should be interpreted taking into consideration some particularities and limitations. Our RNASeq analysis focused on TLR4 and proinflammatory genes, not on other high-ranking but unrelated genes and pathways. Our study was underpowered to detect changes of small magnitude and high variance. We did not perform a time-course analysis in our *in vitro* experiments, which might further show how fibroblast transcription can fluctuate and change over time, as other sequencing analyses have demonstrated (85–87). In our *in vivo* time series analysis, while we were able to measure total CIRP levels in pulmonary tissues and show it is elevated prior to day 14 (**Supplementary Figure 7**), we were not able to measure the levels of eCIRP in the lung interstitial space, which would further clarify the link between eCIRP and pulmonary fibrosis. This model only considered the subcutaneous bleomycin-induced pulmonary fibrosis, which models global inflammatory diseases that cause interstitial lung fibrosis (52, 60, 88, 89). The study of other models can shed light on the role of DAMPs such as eCIRP on other pulmonary diseases causing lung fibrosis.

In summary, we showed that eCIRP induces an inflammatory phenotype in pulmonary fibroblasts, which recapitulates that of lung tissue during the inflammatory phase of bleomycin-induced pulmonary fibrosis. Our results not only shed light on the mechanism by which eCIRP promotes inflammatory pulmonary fibroblasts but also show a possible stage at which this phenotype contributes to the most commonly used pre-clinical model of pulmonary fibrosis. Our study suggests that eCIRP is critical for the proinflammatory fibroblasts reprogramming and may represent a druggable target to stop the progression of pulmonary fibrosis in chronic inflammatory diseases affecting the lung.

## Data Availability Statement

The data has been uploaded to NCBI - accession number GSE178255 (https://www.ncbi.nlm.nih.gov/gds/?term=GSE178255[Accession]).

## Ethics Statement

The animal study was reviewed and approved by Feinstein Institute’s IACUC.

## Author Contributions

SB, MB, and PW designed the experiments. SB and ES performed all experiments and analyzed the data. MB and PW critically reviewed the manuscript. MB and PW conceived the idea and supervised the project. All authors contributed to the article and approved the submitted version.

## Funding

This study was supported by the National Institutes of Health (NIH) grants R01HL076179 and R35GM118337 (PW).

## Conflict of Interest

The authors declare that the research was conducted in the absence of any commercial or financial relationships that could be construed as a potential conflict of interest.

## Publisher’s Note

All claims expressed in this article are solely those of the authors and do not necessarily represent those of their affiliated organizations, or those of the publisher, the editors and the reviewers. Any product that may be evaluated in this article, or claim that may be made by its manufacturer, is not guaranteed or endorsed by the publisher.
