# Supplementary material for: Extracellular CIRP Induces an Inflammatory Phenotype in Pulmonary Fibroblasts via TLR4
Source: Front Immunol. 2021 Jul 23;12:721970. doi: 10.3389/fimmu.2021.721970 (PMC8342891; doi:10.3389/fimmu.2021.721970)
Supplement: Supplementary file 1 [file DataSheet_1.docx]

**Supplementary Figure 1**. **Contribution of selected genes to principal components along the treatment lines**. Principal component analysis (PCA) of the mRNA expression profile of WT pulmonary fibroblasts treated with PBS, 1 μg/ml CIRP, 2 ng/ml TGF-β1, and combination of the two shown with the direction of contribution selected proinflammatory/profibrotic genes. Pulmonary fibroblasts were collected 24 hours after treatments and prepared for RNA sequencing. PC1 and PC2 explained 84 and 11% of the variance, respectively. PC: Principal component.

**Supplementary Figure 2**. **eCIRP differentially enriches TNF-α signaling pathway in the pulmonary fibroblasts**. Differential enrichment profile of WT pulmonary fibroblasts isolated from mice and treated with PBS (orange), 2 ng/ml TGF-β1 (yellow), 1 μg/ml CIRP (purple), and the combination of the two (green) in gene ontology 0033209 (TNF-α signaling pathway). The dendrogram clustering was made based on the hierarchical clustering of samples across all genes for each ontology profile. The heatmap color spectra were normalized across each gene. Z-score color key and histogram of counts presented in the left upper corner.

**Supplementary Figure 3**. **eCIRP differentially enriches IL-6 production-related genes in the pulmonary fibroblasts**. Differential enrichment profile of WT pulmonary fibroblasts isolated from mice and treated with PBS (orange), 2 ng/ml TGF-β1 (yellow), 1 μg/ml CIRP (purple), and the combination of the two (green) in gene ontology 0032755 (positive regulation of IL-6 production). The dendrogram clustering was made based on the hierarchical clustering of samples across all genes for each ontology profile. The heatmap color spectra were normalized across each gene. Z-score color key and histogram of counts presented in the left upper corner.

**Supplementary Figure 4**. **TLR pathway is differentially enriched on day 14 of bleomycin injection in lung tissue.** Examinations of a publicly available gene expression omnibus (GEO) profile: GSE132869. Differential enrichment profile of lung tissues of PBS injected (purple), and bleomycin injected mice on day 14 from the start of injections in gene ontology 0002224 (toll-like receptor signaling pathway). The dendrogram clustering was made based on the hierarchical clustering of samples across all genes for each ontology profile. The heatmap color spectra was normalized across each gene. Z-score color key and histogram of counts presented in the left upper corner. Female mice (~12 weeks old) subcutaneously injected daily either with bleomycin (10 mg/kg/day) or PBS (control). Injections began on Day 0 and were done five times per week for two weeks. Mice were sacrificed on days 7, 14, 21, 28, and 42, and the lung tissues were collected.

**Supplementary Figure 5**. **Cellular response to TNF-α is induced on day 14 of bleomycin injection in lung tissue.** Examinations of a publicly available gene expression omnibus (GEO) profile: GSE132869. Differential enrichment profile of lung tissues of PBS injected (purple) and bleomycin injected mice on day 14 from the start of injections in gene ontology 0071356 (cellular response to TNF-α). The dendrogram clustering was made based on the hierarchical clustering of samples across all genes for each ontology profile. The heatmap color spectra were normalized across each gene. Z-score color key and histogram of counts presented in the left upper corner. Female mice (~12 weeks old) subcutaneously injected daily either with bleomycin (10 mg/kg/day) or PBS (control). Injections began on Day 0 and were done five times per week for two weeks. Mice were sacrificed on days 7, 14, 21, 28, and 42, and the lung tissues were collected.

**Supplementary Figure 6**. **eCIRP induction of proinflammatory cytokines is not dependent on TGF-β1.** Differential mRNA expression normalized counts for TNF-α (**A**), IL-6 (**B**), and IL-1β (**C**) in pulmonary fibroblasts isolated from wild-type (WT) mice treated with PBS (blue), 1 μg/ml CIRP (orange), 2 ng/ml TGF-β1 (green), and the combination of the two (black). At 24 hours after the treatments described, pulmonary fibroblasts were collected and prepared for RNA sequencing.

**Supplementary Figure 7**. **CIRP levels increase on day 10 of bleomycin injection in lung tissue.** Western blot (WB) analyses of CIRP levels in pulmonary tissue mice male mice (~12 weeks old) subcutaneously injected daily either with bleomycin (10 mg/kg/day) or PBS. Injections began on day 0 and were done five times per week mice were sacrificed on day 10, and the lung tissues were collected (4 animals per group). Representative samples shown are from a single blot.
