# Supplementary material for: Extracellular CIRP Induces an Inflammatory Phenotype in Pulmonary Fibroblasts via TLR4
Source: Front Immunol. 2021 Jul 23;12:721970. doi: 10.3389/fimmu.2021.721970 (PMC8342891; doi:10.3389/fimmu.2021.721970)
Supplement: Supplementary file 2 [file DataSheet_2.pdf]

Supplementary Material

Supplementary material 1

Search strategy

embase.com	2445	2410
Medline Ovid	1772	264
Web of science	2539	1233
Cochrane CENTRAL	74	29
Google scholar	200	126
**Total**	**7030**	**4062**

embase.com

('metabolome'/exp OR 'metabolomics'/exp OR metabolite/exp OR 'cytokine'/exp OR 'chemokine'/exp OR 'phosphotransferase'/exp OR 'enzyme'/exp OR 'biological marker'/exp OR 'regulator protein'/de OR 'protein expression'/exp OR 'molecular pathology'/de OR immunopathogenesis/de OR 'virus pathogenesis'/de OR (metabolom* OR metabolite* OR (metabol* NEAR/3 profile*) OR signaling OR regulator* OR omics OR (protein* NEAR/3 express*) OR (molecul* NEAR/3 (patholog*)) OR immunopathogenes* OR cytokine* OR interleukin* OR chemokin* OR peptide* OR kinase OR enzyme* OR biomarker* OR (biological NEAR/3 marker*) OR (virus NEAR/3 pathogenesis)):ab,ti) AND ('urogenital tract inflammation'/exp OR 'urogenital tract infection'/exp OR 'funisitis'/de OR 'intrauterine infection'/exp OR virome/exp OR (('volatile organic compound'/de OR 'electronic nose'/de) AND ('urine'/de OR 'urinalysis'/exp)) OR (((urogenital* OR genital* OR vagin* OR umbilic* OR intrauterin* OR intra-uterin* OR amniot* OR intraamniot* OR urin* OR endometr* OR membrane* OR antenatal* OR ante-natal* OR cervical* OR cervix OR cervicovagin*) NEAR/6 (inflamm* OR infect* OR invas* OR carriage* OR carrier* OR proteomic* OR pathogen*)) OR Amnioniti* OR Chorioamnioniti* OR Funisiti* OR endometrit* OR vaginosis OR vaginitis OR dysbiosis OR virome OR herpes OR ((vocs OR voc OR volatile-organic-compound* OR e-nose OR electronic-nose) NEAR/10 (urin*))):ab,ti) AND ('immature and premature labor'/exp OR 'premature fetus membrane rupture'/de OR (((immatur* OR prematur*) NEAR/3 (birth OR childbirth OR labor* OR labour* OR deliver* OR infant* OR baby OR babies OR rupture* OR neonat* OR parturit*)) OR (rupture* NEAR/3 membran*) OR prematures OR prematurity OR immatures OR immaturity OR preterm* OR pre-term* OR pprom OR prom OR sPTB OR (labour NEAR/3 mediat*)):ab,ti) NOT ([Conference Abstract]/lim) AND [English]/lim NOT ([animals]/lim NOT [humans]/lim) NOT ('systematic review'/de OR 'case report'/de OR ('systematic review' OR 'case report'):ti)

Medline Ovid

(exp Metabolome/ OR exp Metabolomics/ OR exp Cytokines/ OR exp Chemokines/ OR exp Phosphotransferases/ OR exp Enzymes/ OR exp Biomarkers/ OR exp Pathology, Molecular/ OR (metabolom* OR metabolite* OR (metabol* ADJ3 profile*) OR signaling OR regulator* OR omics OR (protein* ADJ3 express*) OR (molecul* ADJ3 (patholog*)) OR immunopathogenes* OR cytokine* OR interleukin* OR chemokin* OR peptide* OR kinase OR enzyme* OR biomarker* OR (biological ADJ3 marker*)).ab,ti.) AND (exp Urinary Tract Infections/ OR exp Reproductive Tract Infections/ OR Chorioamnionitis/ OR ((Volatile Organic Compounds/ OR Electronic Nose/) AND (Urine/ OR Urinalysis/))OR (((urogenital* OR genital* OR vagin* OR umbilic* OR intrauterin* OR intra-uterin* OR amniot* OR intraamniot* OR urin* OR endometr* OR membrane* OR antenatal* OR ante-natal* OR cervical* OR cervix OR cervicovagin*) ADJ6 (inflamm* OR infect* OR invas* OR

carriage* OR carrier* OR proteomic* OR pathogen*)) OR Amnioniti* OR Chorioamnioniti* OR Funisiti* OR endometrit* OR vaginosis OR vaginitis OR dysbiosis OR virome OR herpes OR ((vocs OR voc OR volatile-organic-compound* OR e-nose OR electronic-nose) ADJ10 (urin*))).ab,ti.) AND (exp Infant, Premature/ OR Obstetric Labor, Premature/ OR Fetal Membranes, Premature Rupture/ OR (((immatur* OR prematur*) ADJ3 (birth OR childbirth OR labor* OR labour* OR deliver* OR infant* OR baby OR babies OR rupture* OR neonat* OR parturit*)) OR (rupture* ADJ3 membran*) OR prematures OR prematurity OR immatures OR immaturity OR preterm* OR pre-term* OR pprom OR prom OR sPTB OR (labour ADJ3 mediat*)).ab,ti.) AND english.la. NOT (exp animals/ NOT humans/) NOT (systematic review/ OR case report/ OR (systematic review OR case report).ti.)

Web of science

TS=(((metabolom* OR metabolite* OR (metabol* NEAR/2 profile*) OR signaling OR regulator* OR omics OR (protein* NEAR/2 express*) OR (molecul* NEAR/2 (patholog*)) OR immunopathogenes* OR cytokine* OR interleukin* OR chemokin* OR peptide* OR kinase OR enzyme* OR biomarker* OR (biological NEAR/2 marker*))) AND ((((urogenital* OR genital* OR vagin* OR umbilic* OR intrauterin* OR intra-uterin* OR amniot* OR intraamniot* OR urin* OR endometr* OR membrane* OR antenatal* OR ante-natal* OR cervical* OR cervix OR cervicovagin*) NEAR/5 (inflamm* OR infect* OR invas* OR carriage* OR carrier* OR proteomic* OR pathogen*)) OR Amnioniti* OR Chorioamnioniti* OR Funisiti* OR endometrit* OR vaginosis OR vaginitis OR dysbiosis OR virome OR herpes OR ((vocs OR voc OR volatile-organic-compound* OR e-nose OR electronic-nose) NEAR/10 (urin*)))) AND ((((immatur* OR prematur*) NEAR/2 (birth OR childbirth OR labor* OR labour* OR deliver* OR infant* OR baby OR babies OR rupture* OR neonat* OR parturit*)) OR (rupture* NEAR/2 membran*) OR prematures OR prematurity OR immatures OR immaturity OR preterm* OR pre-term* OR pprom OR prom OR sPTB OR (labour NEAR/2 mediat*)))) NOT TI=(("systematic review" OR "case report")) AND DT=(article) AND LA=(english)

Cochrane CENTRAL

((metabolom* OR metabolite* OR (metabol* NEAR/3 profile*) OR signaling OR regulator* OR omics OR (protein* NEAR/3 express*) OR (molecul* NEAR/3 (patholog*)) OR immunopathogenes* OR cytokine* OR interleukin* OR chemokin* OR peptide* OR kinase OR enzyme* OR biomarker* OR (biological NEAR/3 marker*)):ab,ti) AND ((((urogenital* OR genital* OR vagin* OR umbilic* OR intrauterin* OR intra-uterin* OR amniot* OR intraamniot* OR urin* OR endometr* OR membrane* OR antenatal* OR ante-natal* OR cervical* OR cervix OR cervicovagin*) NEAR/6 (inflamm* OR infect* OR invas* OR carriage* OR carrier* OR proteomic* OR pathogen*)) OR Amnioniti* OR Chorioamnioniti* OR Funisiti* OR endometrit* OR vaginosis OR vaginitis OR dysbiosis OR virome OR herpes OR ((vocs OR voc OR volatile-organic-compound* OR e-nose OR electronic-nose) NEAR/10 (urin*))):ab,ti) AND ((((immatur* OR prematur*) NEAR/3 (birth OR childbirth OR labor* OR labour* OR deliver* OR infant* OR baby OR babies OR rupture* OR neonat* OR parturit*)) OR (rupture* NEAR/3 membran*) OR prematures OR prematurity OR immatures OR immaturity OR preterm* OR pre-term* OR pprom OR prom OR sPTB OR (labour NEAR/3 mediat*)):ab,ti)

Google scholar

metabolomics|metabolites "urogenital|genital|vaginal|intrauterine|uterine inflammation|infection" "immature|premature birth|childbirth|labor|labour|membrane|rupture"|prematurity|preterm|"gestational age"
