# Supplementary figures and images for: Extracellular CIRP Induces an Inflammatory Phenotype in Pulmonary Fibroblasts via TLR4
Source: Front Immunol. 2021 Jul 23;12:721970. doi: 10.3389/fimmu.2021.721970 (PMC8342891; doi:10.3389/fimmu.2021.721970)

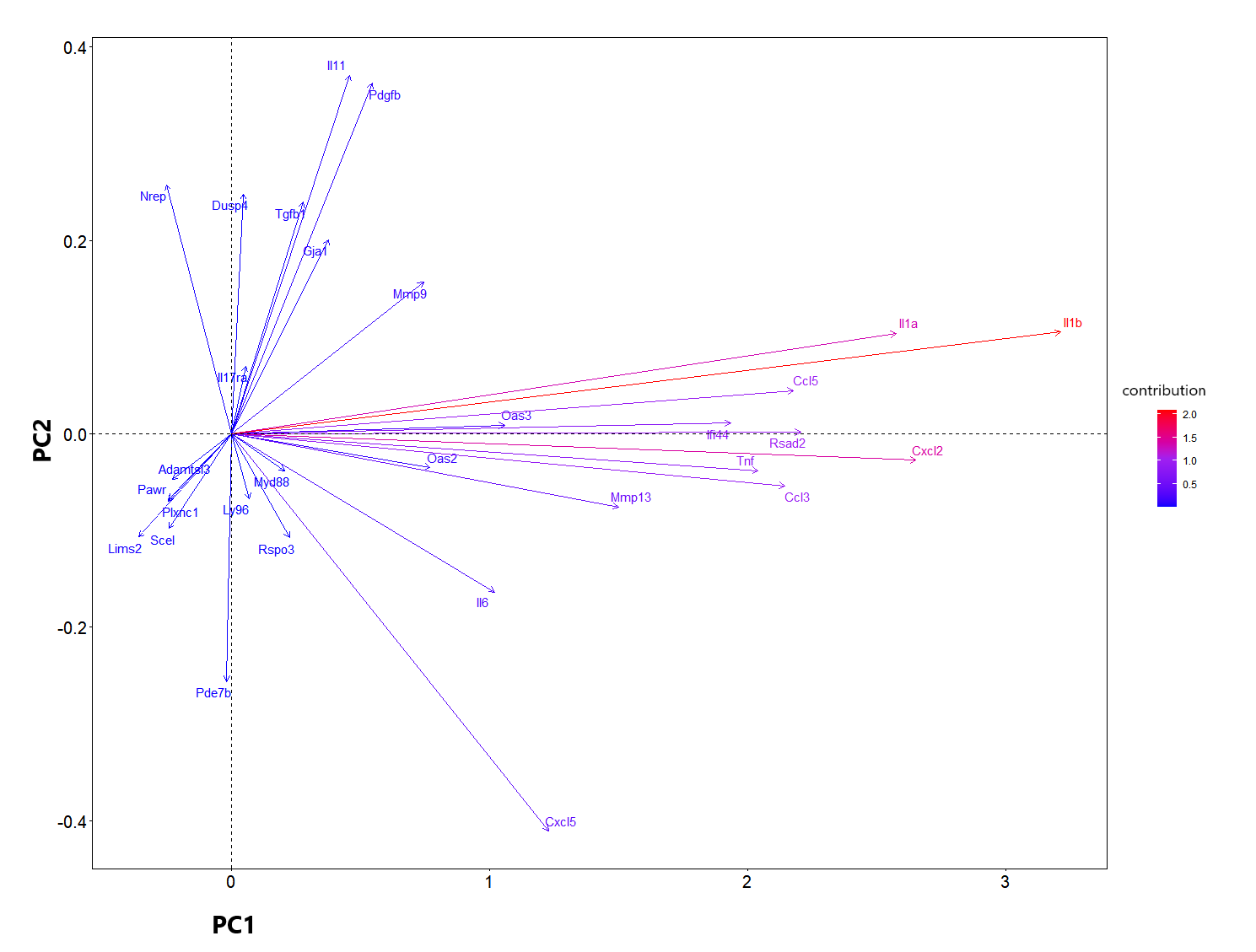

Supplement: Supplementary file 3 [file Image_1.tiff]

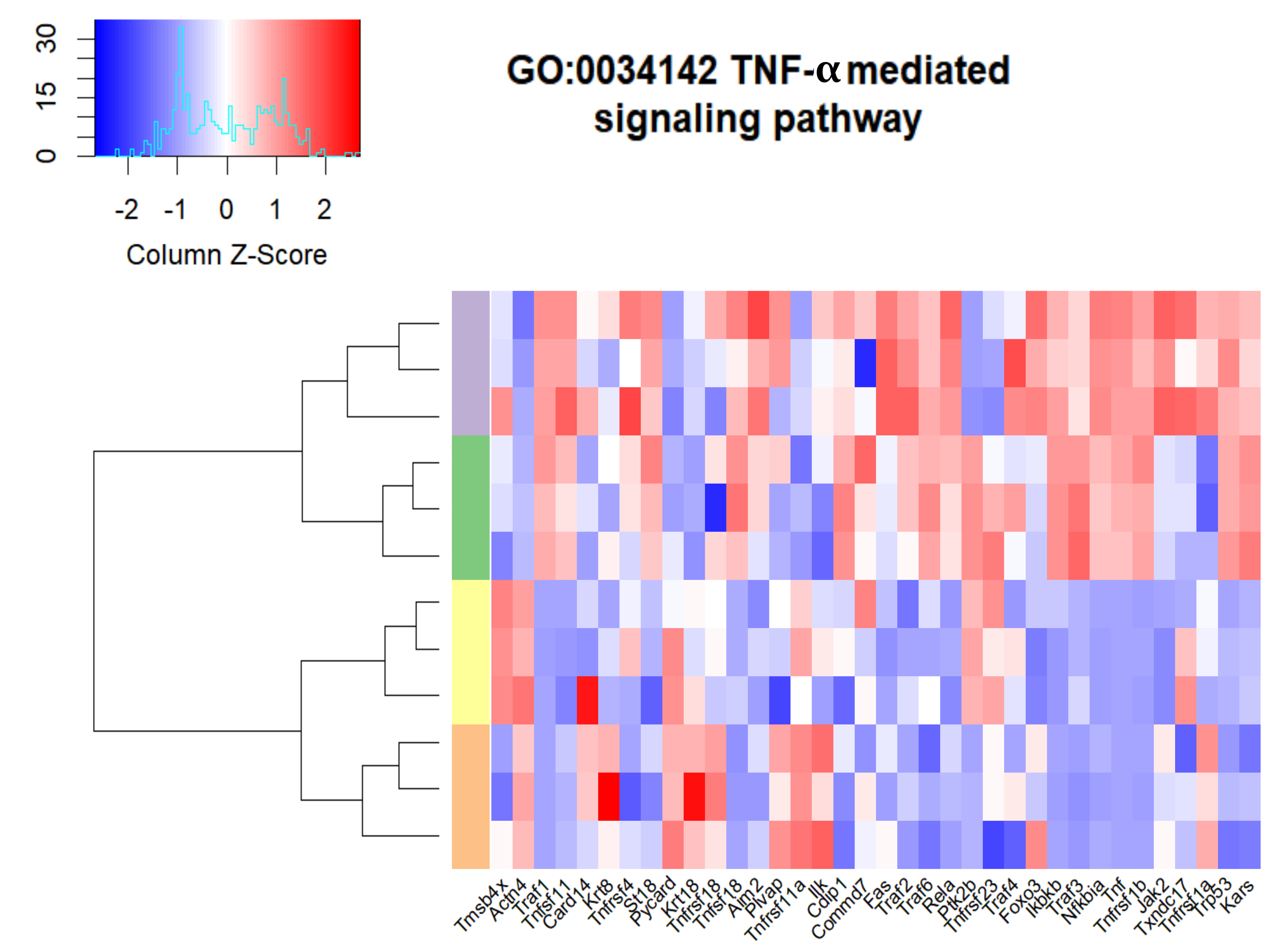

Supplement: Supplementary file 4 [file Image_2.tiff]

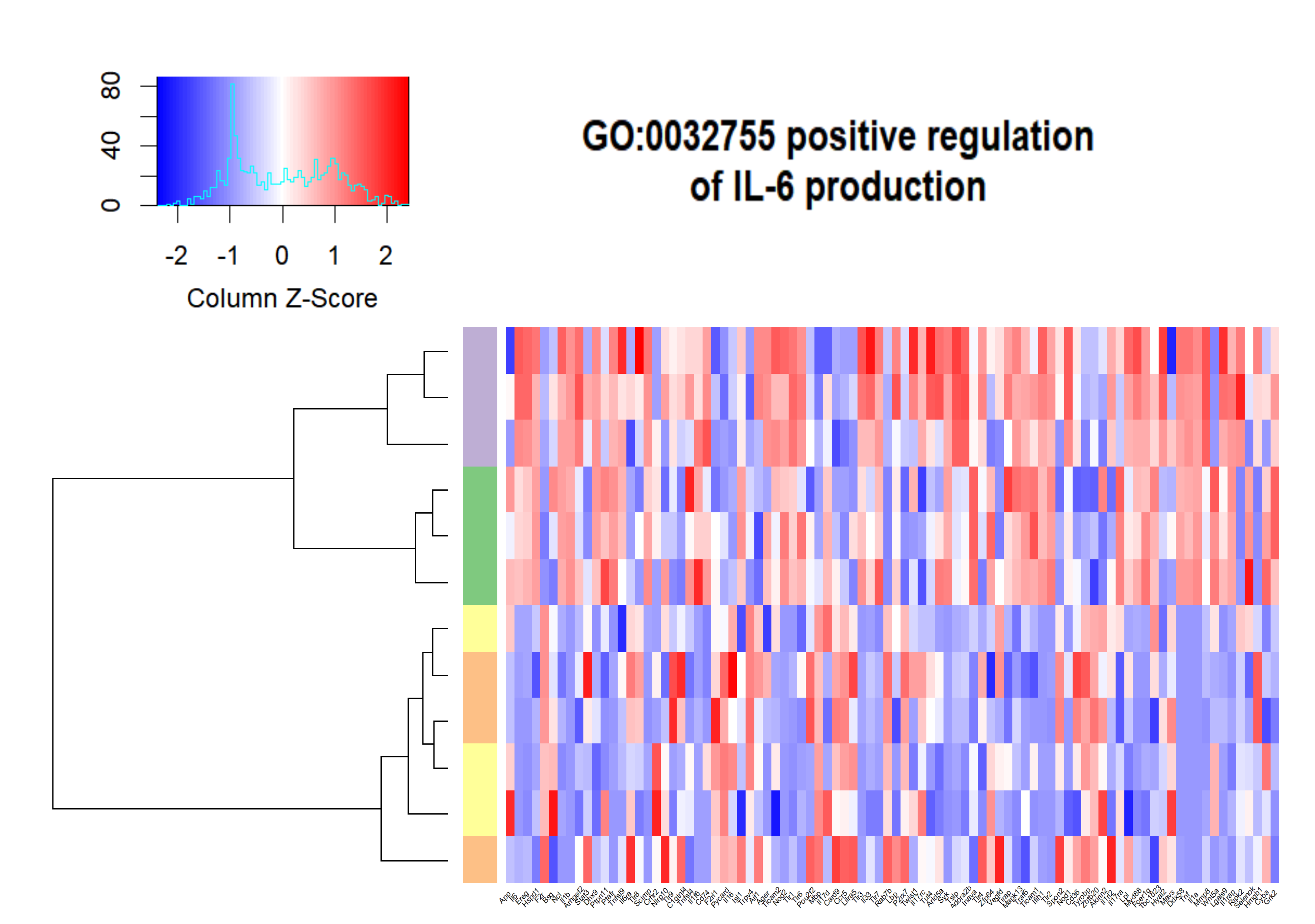

Supplement: Supplementary file 5 [file Image_3.tiff]

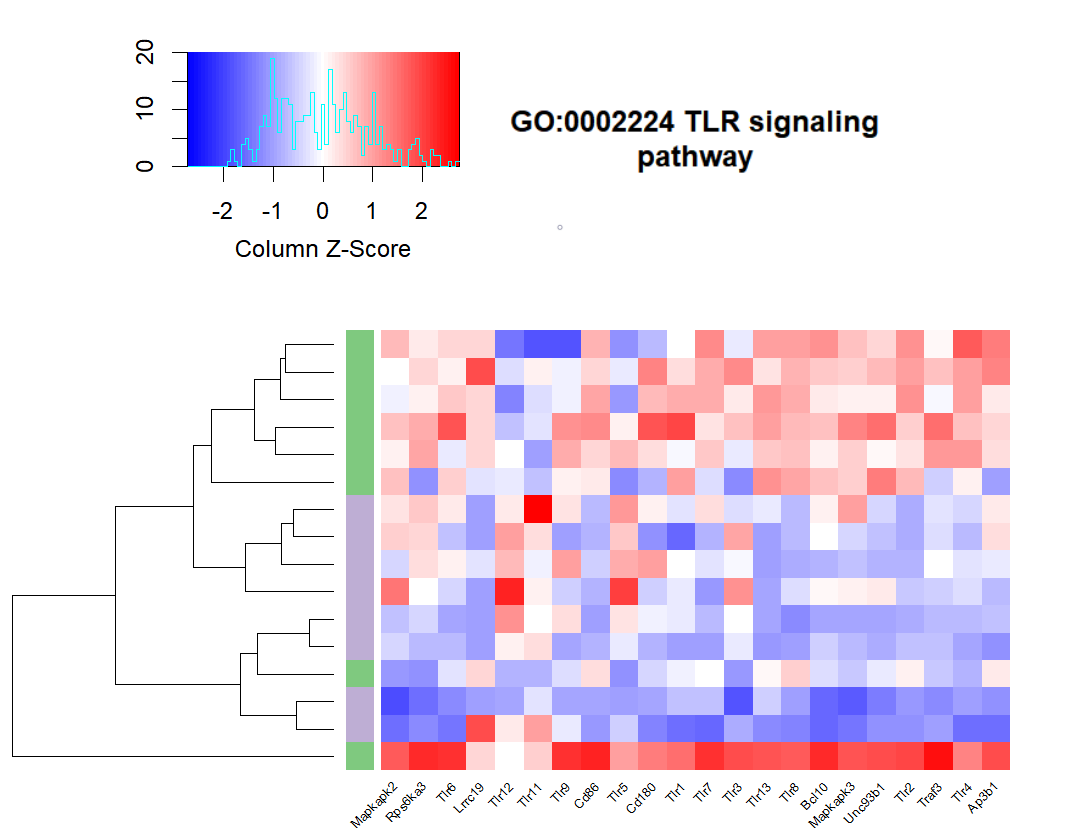

Supplement: Supplementary file 6 [file Image_4.tiff]

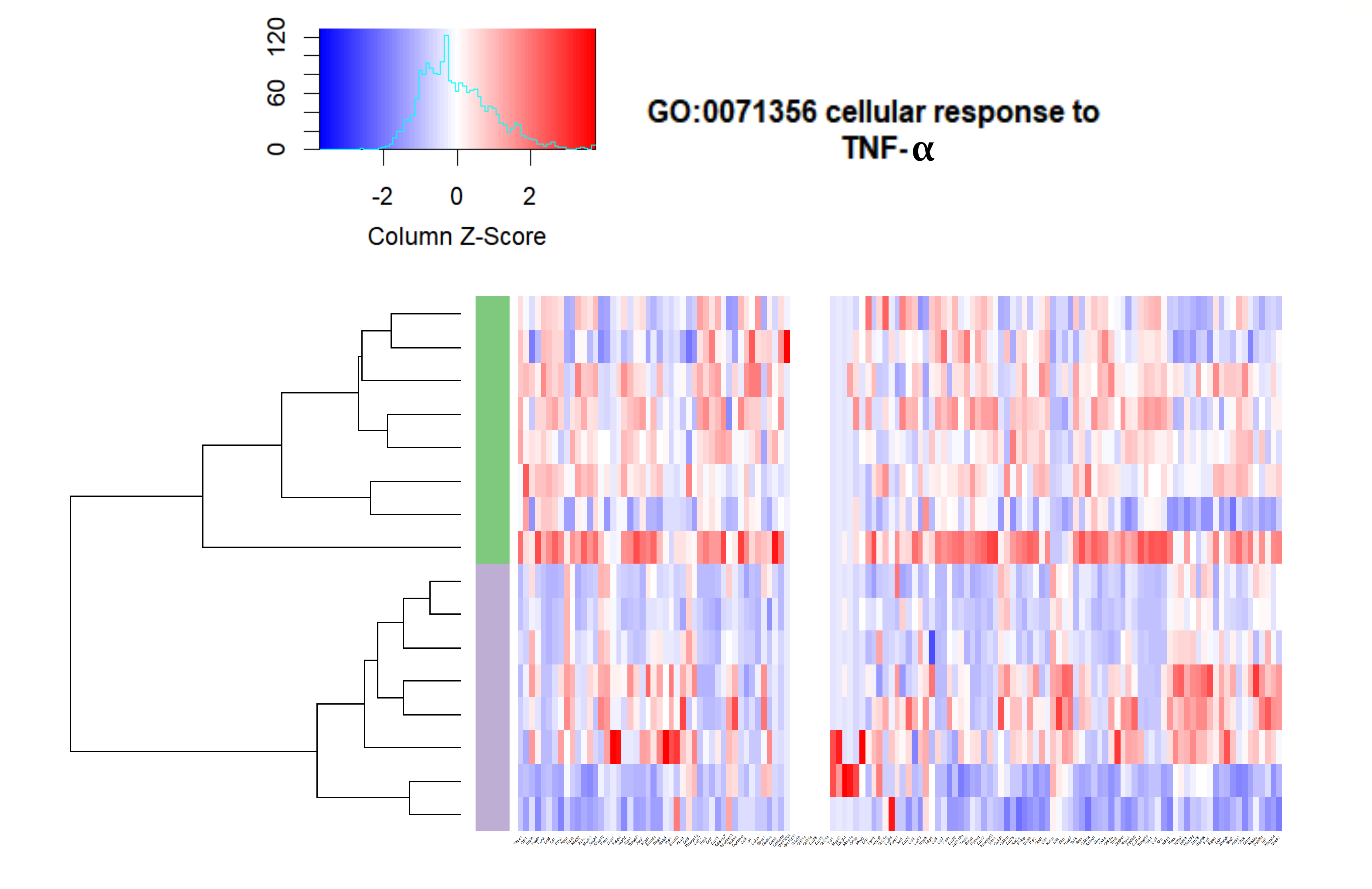

Supplement: Supplementary file 7 [file Image_5.png]

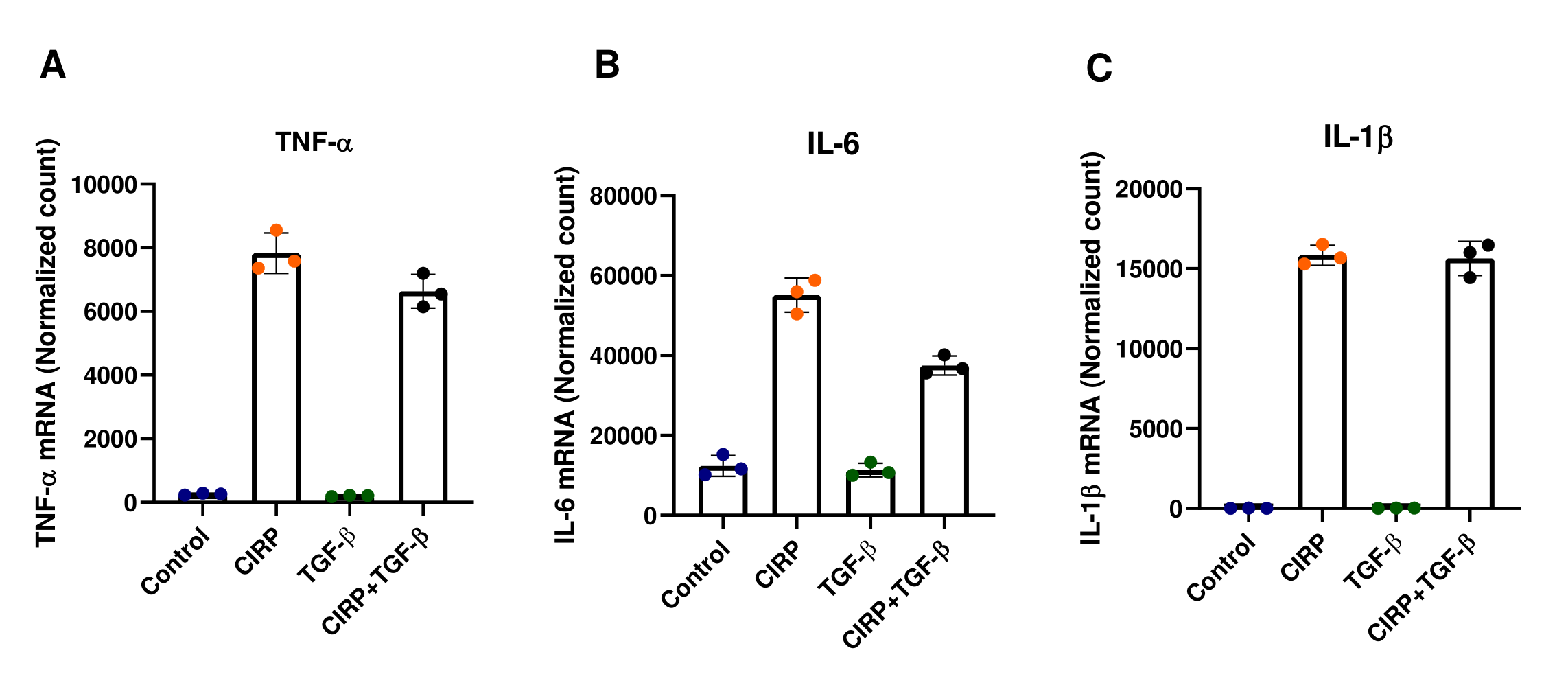

Supplement: Supplementary file 8 [file Image_6.tif]

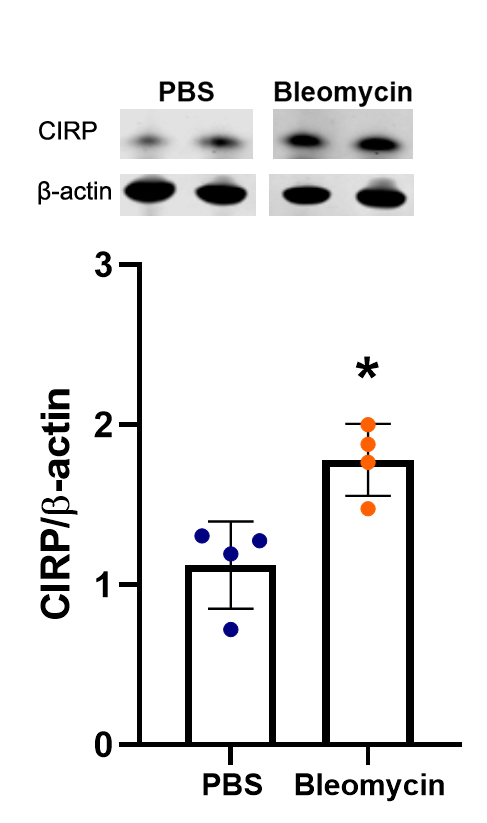

Supplement: Supplementary file 9 [file Image_7.tif]
