# Supplementary material for: Extracellular CIRP Induces an Inflammatory Phenotype in Pulmonary Fibroblasts via TLR4
Source: Front Immunol. 2021 Jul 23;12:721970. doi: 10.3389/fimmu.2021.721970 (PMC8342891; doi:10.3389/fimmu.2021.721970)
Supplement: Supplementary file 10 [file Table_1.docx]

**Supplementary Table 1**. The descriptive statistics of high throughput mRNA sequencing from WT and TLR4^-/-^ pulmonary fibroblasts stimulated with CIRP vs. PBS is described in Figure 1 and 3.

| Gene | Log2fc | LfcSE | W-stat | W-test-p | Adj-p |
| --- | --- | --- | --- | --- | --- |
| WT | | | | | |
| TNF-α | 4.918 | 0.079 | 62.592 | 0 | 0 |
| IL-1β | 9.891 | 0.207 | 47.771 | 0 | 0 |
| IL-6 | 2.149 | 0.174 | 12.338 | 5.65e-35 | 2.31e-33 |
| MD2 | 0.322 | 0.097 | 3.323 | 0.001 | 0.004 |
| TLR4^-/-^ | | | | | |
| TNF-α | -0.026 | 0.07 | -0.376 | 0.707 | 0.997 |
| IL-1β | -0.369 | 0.155 | -2.4 | 0.016 | 0.519 |
| IL-6 | 0.139 | 0.174 | 0.798 | 0.425 | 0.982 |
| MD2 | 0.023 | 0.098 | 0.238 | 0.812 | 0.997 |

WT: Wild type, Log2fc: logarithmic base 2 fold changes on mean likelihood estimates, LfcSE: standard errors of Log2fc, W-stat: Wald statistics, W-test-p: Wald test p-value, Adj-p: Benjamini-Hochberg adjusted p-value
